# “Little things matter!” Exploring the perspectives of patients with dementia about the hospital environment

**DOI:** 10.1111/opn.12153

**Published:** 2017-04-18

**Authors:** Lillian Hung, Alison Phinney, Habib Chaudhury, Paddy Rodney, Jenifer Tabamo, Doris Bohl

**Affiliations:** ^1^ School of Nursing University of British Columbia Vancouver BC Canada; ^2^ Simon Fraser University Vancouver BC Canada; ^3^ Vancouver Coastal Health Vancouver BC Canada

**Keywords:** action research, dementia, practice development, person‐centred practice

## Abstract

**Background:**

Recognising demographic changes and importance of the environment in influencing the care experience of patients with dementia, there is a need for developing the knowledge base to improve hospital environments. Involving patients in the development of the hospital environment can be a way to create more responsive services. To date, few studies have involved the direct voice of patients with dementia about their experiences of the hospital environment.

**Design and method:**

Using an action research approach, we worked with patients with dementia and a team of interdisciplinary staff on a medical unit to improve dementia care. The insights provided by patients with dementia in the early phase shaped actions undertaken at the later stage to develop person‐centred care within a medical ward. We used methods including go‐along interviews, video recording and participant observation to enable rich data generation.

**Aim:**

This study explores the perspectives of patients with dementia about the hospital environment.

**Results:**

The participants indicated that a supportive hospital environment would need to be a place of enabling independence, a place of safety, a place of supporting social interactions and a place of respect.

**Conclusions:**

Patient participants persuasively articulated the supportive and unsupportive elements in the environment that affected their well‐being and care experiences. They provided useful insights and pointed out practical solutions for improvement. Action research offers patients not only opportunities to voice their opinion, but also possibilities to contribute to hospital service development.

**Implications for practice:**

This is the first study that demonstrates the possibility of using go‐along interviews and videoing with patients with dementia staying in a hospital for environmental redesign.

Researchers, hospital leaders and designers should further explore strategies to best support the involvement of patients with dementia in design and redesign of hospital environments.

**What does this research add to existing knowledge in gerontology?**

- This research addresses the knowledge gap by adding voices, opinions and perspectives of patients with dementia about the hospital environment.
- What patients with dementia consider as important has practical implications for making relevant and responsive changes in improving dementia care in the acute settings.

**What are the implications of this new knowledge for nursing care with older people?**

- The care experiences and practical solutions expressed by patients with dementia offer useful insights to guide service development within the acute hospital settings.
- A supportive hospital environment for patients with dementia needs to be a place of enabling independence, a place of safety, a place of supporting social interactions and a place of respect.

**How could the findings be used to influence policy or practice or research or education?**

- Patients with dementia can offer useful insights and want to contribute to research and service development. This research provided empirical support for their involvement and contribution. Leaders, researchers and educators need to explore how to support the involvement of patients with dementia in design or redesign of hospital environments.

## 1 Background

Population ageing is expected to lead to more people living with dementia in Canada and around the world. By 2030, more than 75 million people are expected to be living with dementia worldwide (WHO, 2012). A recent UK study found about half of the older patients (over 70 years of age) admitted to hospital had cognitive impairment (Goldberg et al., 2012). In Canada, older people represent near half (45%) of emergency hospital visits, and many of them admitted for assessment and treatment have dementia (Canadian Institute for Health Information, 2016). Studies have repeatedly reported that older patients with cognitive impairment have worse outcomes compared to those without impairment—including longer lengths of stay, decline in function, and higher mortality rates, while hospital environments have been criticised for being inadequate and insensitive to the needs of older adults with dementia (Clissett, Porock, Harwood, & Gladman, 2013; Dewing & Dijk, 2016; Goldberg & Harwood, 2013). Given the demographic shift in patient population, the workforce and the physical environment of hospitals need to adapt and become more responsive to the changing healthcare needs of the population (Canadian Institute for Health Information, 2016; Parke & Chappell, 2010).

Recent review studies have revealed that there is limited research into the patient outcomes of hospital design, and most of the existing environmental intervention research regarding dementia care has been conducted in residential care settings (Chaudhury, Hung, & Badger, 2013; Fleming, Goodenough, Low, Chenoweth, & Brodaty, 2016). Another significant problem is the lack of knowledge about the first‐person perspective from patients with dementia. There is misconception and stereotype that people with dementia cannot communicate their views (Cowdell, 2010), despite an increasing body of research illustrating that people with dementia have important insights to offer and can contribute to knowledge production in meaningful ways (Phinney, Chaudhury, & O'Connor, 2007; Phinney, Dahlke, & Purves, 2013; Sabat, 2002). Swaffer (2014) rightly argued that doing research about dementia without involving people with dementia cannot provide a true portrayal of salient issues for this group and ignoring the experiential knowledge of people with dementia hinders the validity of research evidence. Research evidence has shown that there is incongruence with what care providers report and what is observed about the experience of people with dementia (Innes, Kelly, Scerri, & Abela, 2016). Swaffer (2014) argued the literature has been giving wrong descriptions of people with dementia and has created misconceptions. She explained:

Much of the published research is biased through the use of family carers as the main cohort group, or having them in attendance when people with dementia are interviewed, and so the carer voice remains the same voice in the dementia literature. (p. 710)

In addition, the white paper by the Dementia Action Alliance (2016) located in the United States pointed out that professionals might have a limited perspective about the lived experience of people with dementia. Care providers in acute care settings tend to have priorities in medical procedures, infection control, risk management, and length of stay. Patients may have different values and other priorities. Research that provides understanding of patients’ needs and experiences from the perspectives of patients with dementia is needed to inform meaningful changes in hospital environments. The issues that patients with dementia consider as priorities ought to have practical implications for effective allocation of resources, thus making relevant and responsive changes. Given that patient stories can be pivotal drivers for changes in the broader healthcare context (Bate & Robert, 2007), patients with dementia should be supported to give their voice in research and be included in conversations about the hospital environments and services that affect their care and experiences.

Overall, in the literature, there is a growing recognition of the need for more inclusive designs for people with dementia and a better dementia‐trained workforce to ensure hospital stays do not add disabilities and compromise well‐being (Francis, 2013; Innes et al., 2016). Researchers and practitioners have reported that the traditional designs of acute hospitals are not responsive to the specific needs of people with dementia. Studies reported that unclear signage, poor lighting, clutter, and a lack of space for family visits, and opportunities to engage in meaningful activities are common problems in hospital design (Digby & Bloomer, 2014; Hung et al., 2014). Concerns have been voiced related to the experience of people with dementia in hospitalisation who are experiencing high distress; at the same time, nurses are constrained in attending to that distress by structural environmental factors as well as a lack of the staffing support required to provide person‐centred care (Cowdell, 2010). Moyle, Borbasi, Wallis, Olorenshaw, and Gracia (2011) found that the acute care environment influenced staff attitudes with regard to the care of people with dementia, leading to excessive monitoring of patients and less emphasis on meaningful interactions between staff and patients.

The challenges of dementia care in acute hospitals are complex; therefore, it is necessary to examine the processes through which the care experience of patients with dementia may be impacted by both the physical and social environments in dynamic interaction. For the purpose of this study, physical environment refers to the built features in the environment, such as wall colour and lighting; social environment involves human factors, which include care practices in a relational context.

## 2 The Person‐Centred Care in Acute Care Study

This study is part of the *Person‐Centred Care in Acute Care Study*, which is an action research inspired by a quality improvement project by Waller, Masterson, and Evans (2016). Waller et al. (2016) reported positive effects on patients with dementia (e.g. reductions in falls and the use of antipsychotics), by making simple environmental changes in a surgical unit (e.g. enhancing colours and adding comfortable seating areas for social interactions). Although this quality improvement project offered good support for environmental strategies, it did not report details of the methodology and the processes of stakeholders’ involvement. It is unclear whether patients with dementia were involved, and if so, what they contributed and how the inquiry took place.

Given the identified gaps in the field of dementia research in acute care, our research began with exploring the experiences of patients with dementia—their firsthand perspectives of the hospital environment. In particular, [first author] used go‐along interviews to engage patients with dementia to identify: what and how specific environmental attributes impacted their care experiences and what they wished to see as improvements to the issues they identified. This study reports data related to the first phase of the larger research project that aimed to make physical and social environmental changes for improving the care experiences of patients with dementia in a medical unit of a large urban hospital. The research was designed so that insights provided by patient participants in phase one would inform actions for developing physical environmental changes and staff education in phase two, and serve as a part of the base for assessing impacts made by the changes in phase three. The processes and findings for phases two and three will be reported in future papers.

## 3 Design and methods

### 3.1 Theoretical grounding

The study was part of an action research study underpinned by critical social theory (Habermas, 1984) and interpretive approach (Gadamer, 2011). The interpretive approach was helpful in making sense of patients’ narratives to convey understanding. The participatory perspective of action research emphasised the value of researching “with” rather than “on” people (Bradbury, 2015)—a particularly salient approach for people (such as patients with dementia) who are among the most marginalised in our society. In the study, patients with dementia were not treated as passive subjects to be studied, but as active agents who had important contributions to make. This approach embraces Habermas's theory of communicative action (1984), which suggested the conditions necessary for egalitarian communication include freedom from manipulation and domination of power. In action research, there is a deep respect for the rights of people to have their say in how knowledge is generated about them. As Kemmis argued (2008), the inquiry itself in action research is a form of political action. In this study, patients with dementia in the medical unit were invited to tell their stories, experiences and suggestions for making improvements in the hospital environment. Through the processes of elicitation and recognition, patient participants were given a sense of power and legitimacy of their knowledge about the environment and expertise of their lived experiences.

### 3.2 Setting

The study took place in a 31‐bed medical unit of a large urban hospital in Canada. The unit provides assessments and treatments to a general population of patients requiring medical and nursing care. Typically, about a quarter of the patients have dementia and the common types of diagnoses include cerebrovascular accident, heart or lung diseases, sepsis, fall injuries and confusion. Patients stay in the unit for a varied length of time, ranging from a few days to several months.

The unit has a layout with two, long double‐loaded corridors (each corridor has patient rooms on both sides). The nursing station is located where the two corridors meet and is situated between two locked entrance doors. Many patients like to sit around the nursing station to watch nurses doing their work. The traffic through the two entrance doors often triggers conflicts with patients trying to go off the unit, and staff members are under constant stress trying to monitor and control the exit. Walls are painted a pale neutral colour and handrails are in light brown, which is similar to the wall colour. Linen carts, beds, wheelchairs and other equipment line the corridors, which at times limits access to handrails. Various paintings are hung on the walls, but they tend to be obscured from vision by the tall linen carts and staff signs (e.g. infection control, violence alerts). A television for patient use is placed at the entrance to a corridor that is close to the nursing station. Meals are brought to the unit in trays and patients eat in bed. Most of the rooms have three beds, although a few are single‐bed rooms that are usually reserved for patients with infections that require careful isolation procedures.

### 3.3 Participants

Purposeful sampling (Patton, 2015) was used to identify patient participants to gather in‐depth and meaningful insights. Nurses who knew the patients on the unit provided assistance in recruiting the patient participants with diverse characteristics to maximise variation. Among the five participants, three were men and two were women, with an age range of 65–84. All participants had a diagnosis of dementia, including Alzheimer's disease, vascular dementia or an unspecified subtype of dementia. They had a wide range of functional abilities and difficulties. Some were independent and steady in walking; others were wheelchair or walker users. We included patients who were identified as having behavioural and psychological symptoms of dementia, including agitation and aggression. Some were unsteady in walking and had struggles with wayfinding. Some had more difficulties in word finding; others were skilful in articulation. Their ethnic backgrounds were also diverse, including descendants and immigrants of European, American and Asian origins. Participants had varied education levels and occupational backgrounds, including an artist, a photographer, a fashion buyer, an odd job worker and a business owner. The decision of selection was based on the logic of seeking information‐rich cases (Patton, 2015). The small sample allowed us to yield not only a deeper understanding of patients’ experiences but also commitment and actions of staff to make change as the compelling stories were revealed.

### 3.4 Data generation

Ethnographic methods, including go‐along interviews technique and videoing (Iedema, Long, Forsyth, & Lee, 2006), were used to support patients with dementia to voice their views and experiences about the hospital environment. During each conversational interview, patient participants were asked to take the lead in topics that they considered as important and wanted to discuss while taking for a walk together in the corridors of the medical unit. Following the participant, the first author used a small, hand‐held camcorder with a narrow‐angle lens focused on the participant or on particular features of the environment. Instead of relying on memory recall, participants were invited to talk about what they saw, heard and sensed in the immediate environment while moving through the unit. When objects or artefacts were visually accessible and events were taking place in the environment, participants with dementia were better supported to tell stories about experiences and express their views, an approach that has been used in previous studies (Hubbard, Tester, & Downs, 2003; Hung, 2015). At the same time, experiencing the environment with the participant together made it easier for the researcher to understand and make sense of meanings that the participant was trying to convey. In order to bring focus to the research topic, occasionally, the researcher asked what the participant liked or did not like about a specific feature of the environment and the associated reason. What could be changed to make the environment better was also inquired. Each patient participant was interviewed twice, with each session lasting around 30 min. A few interviews involved a one‐on‐one walk, while others involved two participants at the same time, based upon the request of the participants. All narratives in the videos were transcribed verbatim, while the visual recordings helped to capture both verbal and non‐verbal expressions, as well as materials in the environmental context. Field notes were taken to record immediate feelings, thoughts and questions that could require further clarification in the data collection.

To gain a background understanding of the everyday activities in the studied environment, participant observations were conducted in the corridors of the unit. A total of 20 hr of observation was conducted during weekdays and weekends over a 3‐month period (January–March, 2016). Participant observations allowed the researcher to get close to the experiences of the participants, and develop a connection with the patients in the field while observing the general activities (Emerson, Fretz, & Shaw, 1995). During the observations, the researcher either sat in a chair or stood in a corridor, sometimes conversing with the staff, patients and families. Field notes were written in a notebook to record details of how the patients were interacting with other people and the physical environment. Exquisite attention was paid to small mundane activities and striking events (Katz & Alegria, 2009), as well as reactions of patients and staff as they unfolded in situated moments.

### 3.5 Data analysis

Drawing on the interpretive approach based on Gadamerian hermeneutics (Fleming, Gaidys, & Robb, 2003), the data analysis focused on understanding people in context, which means interpreting what and how specific environmental attributes affected the care experiences of patient participants. Data analysis was iterative and conducted with data collection. Three broad analytical phases were involved, and these phases were carried out in a cyclic mode throughout the analysis process, requiring repeatedly return to data and the coding to refine the theme development. For a preliminary analysis in the initial phase, the first author began with watching all visual data and reading of the transcripts and field notes several times to gain a sense of the whole. The visual data, transcripts and field note were pooled and coded in NVivo 11 to facilitate analysis. Both inductive and deductive approaches were used. While the data set was primarily coded inductively, concepts based on the literature in environmental design for dementia care were also used for deductive coding. For example, “nothing to do” was an inductive code used to capture segments of narratives. “Colour contrast” was a sensitised concept, a deductive code, informed by the literature. The process involved going back and forth between the data and the literature.

Understanding people in context also means coming to a social agreement through dialogue (Gadamer, 2011). Therefore, the second phase involved two co‐investigators, and the first author (JT and DB). Reviewed the key points in the videos and transcripts together in biweekly research meetings where individual interpretations and tentative analyses were compared, challenged and discussed to bring a more clear focus to be further developed. Collectively, particular video segments were selected to illustrate key themes.

Patients were provided opportunities to view their own footage. Three of them viewed their footage; the other two chose not to view. When videos were played back to patient participants, they tended to make more comments on their appearance in the film, rather than the content of the data. With permissions given by patient participants, video segments and extracts of transcripts were reviewed with frontline staff and leaders in focus groups/reflexive sessions.

The third phase of analysis in nine video reflexive sessions provided opportunities for the team to discuss the issues patients encountered and possibilities for future actions. The discussion focused on what could be learned from the patients’ stories shown in the videos. A total of 50 staff in the team attended the groups, including nursing staff (30), physicians (15), and allied health, including staff in physiotherapy and occupational therapy (5). The overall analysis process involved moving from considering the parts (what patients said) to the whole (what happened in the background and context) and back to the parts. With the expanded understanding of the whole, meaning of the parts can be widened (Fleming et al., 2003). For example, discussion with staff revealed how stereotypes of dementia and physical appearance had caused misunderstanding of what a tall patient participant meant in his language and behaviours. Details of life history and stories of care interactions provided a broader lens to understand his narratives.

Through an iterative process of discussions within and between staff groups, themes were developed and modified and refined based on agreements. The analytic approach was a social process, which took multiple cycles and involved different people to view the data multiple times in different ways. The two co‐investigators (JT and DB). Participated in the video reflexive sessions with the staff. Although the first author initiated the analysis, the process was supported by continuous discussions with co‐investigators and supervisors of the research committee through regular research meetings. Thus, the final themes developed were a shared interpretation of the researchers and participants involved.

### 3.6 Ethical considerations

Ethics approval was granted from both the University Research Ethics Board and the local health authority. The research followed current consensus guidelines, treating consent as an ongoing process, seeking assent and respecting any dissent of the participants (Black, Rabins, Sugarman, & Karlawish, 2010; Dewing, 2007). Written consent was initially obtained, and verbal assent was sought before and during each interview session to remind participants about the purpose of the research and their right to withdraw at any time. A family member signed the participant information and consent form in cases where the participant was unable to do so. In the consent form, participants were given options to allow the researcher to use video or not at each interview. Note taking and audio recording were offered as alternative options. I booked appointments with each participant to do the interviews, so they have time to consider the options. In the study, no patient participants declined videoing. During each interview, I checked and rechecked participant's verbal and non‐verbal response in changing situations. For example, 1 day, a participant told me a story with videoing. A few minutes later, she decided that she wanted that story to be deleted. I respected her wish and deleted the story in front of her.

### 3.7 Ensuring credibility and quality

The quality of action research hinges on the participatory processes and the production of actionable knowledge to move towards making improvement of human experiences (Bradbury, 2015). In this study, participative values were enacted through collaborative working with patients with dementia, a seldom‐heard group (Swaffer, 2014). Multiple groups of stakeholders were involved in multiple steps of data analysis to ensure that the themes were the best possible representations of the data. Direct quotes from the narratives were used to help readers make judgements of the fit of representations. As Gadamer (2011) has explained, there is no single interpretation that is universally true, and understanding can only be achieved by consensus through dialogue (between people or between reader and text). To ensure the scientific rigour of the study, we performed an iterative hermeneutic process systematically, using gathered data, emerged interpretations and available literature to make a coherent set of themes.

## 4 Findings

**Figure 1:**
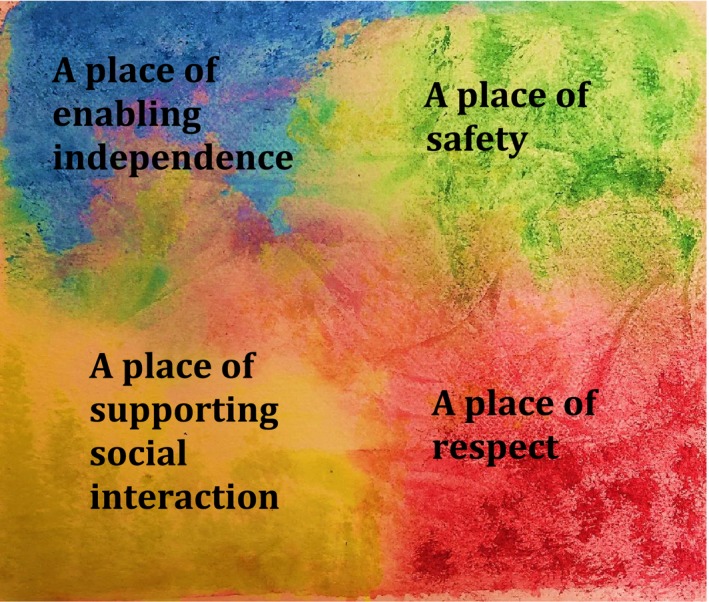
Key aspects of a supportive hospital environment

Data analysis yielded insights about the key aspects of the hospital environment's impact on the care experiences of patients with dementia, and what patients with dementia wished to see as improvements related to the issues they identified. The characteristics of the physical and social environments were described to impact positive and negative care experiences of people with dementia. Here, we present the findings as four interlinked themes. Firstly, *a place of enabling independence* points to the importance of positive engagement of the brain and body. Secondly, *a place of safety* means not only being physically safe, but also feeling emotionally and psychologically safe. Thirdly, *a place of supporting social interactions* speaks to the essential need to have opportunities for human connections. Fourthly, *a place of respect* describes the central concern that patients with dementia need to feel socially included and have their rights respected in the hospital (Figure 1).

### 4.1 A Place of enabling independence

For the participants in this study, being a patient in hospital meant a loss of independence. The reduced opportunities to perform everyday activities affected their sense of autonomy. They wanted the hospital to be a place of enabling independence rather than disempowerment.

Participants described that the hospital should afford patients opportunities to do familiar things that they always enjoy and consider as purposeful. The meaningful activities serve a vital function to keep the brain and body active, which contributes to health, healing and well‐being. For the participants, simple day‐to‐day activities such as going for walks or meeting someone for conversation were essential in promoting a feeling of independence. One participant said, “I can't sit and do nothing at all. Everyday, I make my bed. I always come out to find someone to talk to. It's nice that if you are capable of doing things. It's just the way I am. I'm very independent. I do everything. It's very, very important.”

The biggest barrier that stopped some patients to come out to the corridors to walk was the clutter of linen carts, medical equipment, hallways beds, etc. The combination of simultaneous loud sounds from other patients, the staff and non‐stop multiple alarms ringing made the environment confusing and distracting. Busy and crowded signage on the walls concerning infection practice and other reminders/notices made the place even more over‐stimulating. One participant referred to the clutter thus: “Chaotic clutter makes the brain feel getting overloaded by too many stimuli, overly charged with electricity if you know what I mean.”

One patient suggested small practical changes could make a difference, “The corridor could look very inviting if it's done properly with shelving and if it's up nicely.” She went on to offer ideas of how to organise shelving in aesthetically pleasing styles suggesting that the corridor would look much calmer and be twice as wide if the clutter was put away in storage and if the confusing signage was replaced by beautiful artwork.

An environment that is difficult to navigate and comprehend can have a negative impact on independent functions. According to the participants, wayfinding was a common concern, which made independent navigation challenging if not impossible. Two patients commented that the identical room doors and non‐distinguishable hallways made finding their room difficult. One of them said, “Especially when I am tired, room numbers on the wall was not always helpful. I don't really see them and I don't find number meaningful for me.” Another patient echoed, “Personally, at times I get a little stuck with the number too, remembering the numbers.” One patient mentioned that different colours should be used in each wall so it would help patients to know they were in right or wrong corridor for their room. She recommended: “The color can be a contrast; perhaps it can be the same but then a deeper tone.”

Another patient suggested that bright colours should be used to encourage people to get out of the bed and come out to walk. She explained,

In a hospital, you wouldn't feel quite so much in it if you had some color around. People staying in bed all day could get stuck in thinking about their situations and become very worried and depressed. The use of color can help uplift a patient's mood and emotions. Thoughtful variations of color and art painted on the wall could make the place look more homey. I think that half the walls could be one color and half another color…color would make the hospital feel more comfortable. I think it could be fun colors, all different colors. People feel they are at home a little bit, not so much stuck, dying or whatever. It would give them a nicer feeling about where they are.

For this patient, colour can be powerful in terms of stimulating senses, shaping the ambience of the place, reducing anxiety and worries, encouraging mobility as well as improving mood.

The lighting in the corridors was identified as a significant contributor to the use of space and patient mobility, especially for those who had eye conditions or were visually impaired. One participant who had a common age‐related eye disease refused to go into dark areas in the hallways. He also seemed to have difficulties with glare on the floor. In the interviews, he tended to walk around the glare spots on the floor, which could increase the risk of falling. Interestingly, observations showed that many patients did not use the handrails on the walls, and this could be for two possible reasons: the clutter of clinical equipment and hallway beds often blocked access to the handrail, and the colour of the handrails did not contrast with the background (both were in neutral colour). To encourage independent walking and mobilisation, a participant suggested the need to have seating areas in the corridors. “Because people get tired when they walk down the hallway, just putting a simple piece of furniture at various places could help people rest and feel safe to walk.”

### 4.2 A place of safety

Many participants spoke of the need to be in a place of safety as a priority, with this often described as a psychological need to feel safe emotionally, not just physically. Psychological safety is associated with the physical features and relational aspects of the hospital milieu. Feeling emotionally unsafe with other patients seems to impact psychological safety, which has implications for increasing anxiety and reducing abilities to cope with perceived threats. When asked what would help them to feel safe in this environment, patients mentioned how some aspects of the aesthetics and practicality of the environment were significant to their feelings of safety. For example, a patient, who had difficulties in visual‐spatial perception, told the researcher that he felt threatened when people moved too quickly or were too close to him. “I don't feel right with those people coming by. Boom! Like this, all the time.” He also felt unsafe to go to some areas in the corridors that were cluttered with equipment. The noise of patients calling out or crying also frustrated him. “See how they cry? It's common. I'd rather stay away from them because I'd probably smash their head.” It was evident that the environmental features can have significant impacts on the feeling of safety emotionally and psychologically. Also, feeling safe can be just important as being physically safe for patients with dementia. During the interview with the same patient on one occasion, I noted that he became unbalance as he flinched and pulled himself away from a patient who got close to him. He was very sensitive to any movement in space and felt he constantly needed to protect his personal space. Similar scenarios of people encountered in busy and crowded traffic often were triggers of conflicts in the corridors. A participant described how overcrowding with equipment in the corridors could evoke feelings of danger and actual risk:

The brain needs to relax to function. Feeling stressed definitely does not help. Well, here's one example right by us. This is for blood pressure. It's a danger to people who are not as conscious up there [pointing to the brain]. I mean that's very unwise to have it where it is. You can't rely on somebody walking and necessarily stopping if their minds are somewhere else or got caught up with too many things; then they're not focused on their walking space.

The same patient further explained that the abilities of people with dementia might be reduced by changes in their attention and concentration. “I find it very hard for me to concentrate on anything when too many things were coming to my brain at the same time, which makes me feel exhausted.” Another patient commented that a tidy and organised place would show respect for patient safety. In the video review sessions, staff in the team collectively commented that more attention should be paid to consider the needs of patients who are older, frail, and have cognitive or other functional difficulties, instead of organising the supplies and equipment only for staff's convenience.

A variety of views were expressed on what contributed to feeling safe or unsafe and how best to enhance a sense of safety in the ward. One patient said, “We have a person who keeps going up too close and is always moving so fast in the hallways…she could easily push you over.” Another patient added, “There is a very confused guy who goes into other people's bed at night. It is vitally important to have enough nurses around… The nurses here are quick to act to keep everyone safe.” Another patient nodded her head and agreed with the statement that having nurses available and nearby would help her feel safe. Also, one patient commented about the need to have places to get away from the stress of noise. “It would be nice if there is a place that I can sit quietly with a cup of tea.”

### 4.3 A place of supporting social interactions

In the observations, a sharp contrast was seen between the experiences of the patients and those of the staff. The traffic in the corridors was heavy and fast paced, and the general ambience of the unit was dominated by clinical activities. The housekeeping staff worked non‐stop, sweeping and cleaning. Some of the nurses did not always walk, but hopped and ran, and the laboratory technicians were frequently pushing the diagnostic equipment through. The patients, however, sat for hours and had nothing to do. The participants expressed their feelings of boredom while the staff was under time pressure to get their tasks done. One staff worker said, “For us, we're like running around, busy. For them, it's like, what are we doing? There's nothing to do.” Another staff added, “We should have room for activities, a little area for coffee or tea. The patients feel so bored here.” The issue of profound boredom was a consistent theme expressed by all participants. One patient explained having nothing to do could become a stressor affecting health:

I guess one of the stressors involved with being in the hospital is you don't have too much to do. The hospital is a very boring place to be because nobody does anything—zero. There's nothing for anybody to do. They just sit around and hope for the best. People need positive distractions to allow them to redirect their thoughts to good memories about themselves or good things about life.

The patients reported that activities are important for health and well‐being as they can support the feeling of social connection, purpose and a sense of belonging in the world. Positive conversations are needed in social spaces to spark positive convivial emotions for the patients.

One patient shared his experience of being ignored, which was perceived as demeaning and offensive. When he was asked about how he found the hospital place where he was staying. He responded: “Too many people here, they don't look either, one way or the other. And I don't like it.” For him, not being acknowledged and not included in a conversation meant people did not care or value him for being there. Social interactions are considered important because they not only provide social and cognitive stimulation to help maintain function, but conversation affords opportunities for expressions of personal identity and a sense of being accepted as a member of a group (Ryan, Bannister, & Anas, 2009). For the participants interviewed, it was evident that what was really important in their care experiences was to be treated as someone who mattered. Participants spoke of the importance of being accepted as a valued person in the world. One patient shared her thought in this way:

An adjustment to being in the hospital is, you know, has an effect on the patient. It's very hard for me, not just physically but also emotionally. It takes time to adjust. Hopefully, I make a few friends through the process, so I don't feel so alone.

She went on to suggest a possible solution:

I think the hospital should have volunteers to come in to do things with patients. Have a room where people can do what they're good at; paint or they like to do macramé. I don't like sitting in my room all the time, so often I'm out here looking for someone to talk to. I'm a friend of [a patient's name], yeah. I like her. We talk, we can relate to things that happen in the city. It's interesting to hear her point of view.

The importance of maintaining a sense of normalcy and continuity was evident in the narratives of the participants. Participants also insisted that patients need to have opportunities to engage in familiar activities, things that they always enjoy and like to do to maintain a sense of identity, express who they are and increase self‐esteem.

Another patient suggested having a space to do programmes of activities would be helpful because involvement in activities offered patients a way to express their emotional and psychological needs, which was not always straightforward for some patients:

Activities offer a way to express oneself. You might find out that some patients suppress their problems because they don't talk about it or not able to say it out. Rather than hold in some areas of difficulties that relate to their psychological problems and frustrations, through art or music, people would have a venue to express what they're feeling, what they would like to say.

Another patient added, “Yep, activities would help nurses to know what's more important to a person and why.” This is a very insightful comment. More knowledge of a person's life permits the nurse to incorporating patient's value and belief into the planning and delivery of care.

### 4.4 A Place of respecting patients’ right

A common theme voiced by the participants was the concern of social exclusion, and that patients should have their rights respected in the hospital. Participants spoke of how their rights to autonomy and control deserved respect. Having their viewpoints disregarded has led them to feel devalued and disrespected. In one case, a patient shared his experience of being restrained and how it made him feel sad and powerless.

I spend my day being tied up in this chair most of the time. They worry about I fall. The first time I fell because I was not used to the kind of floor here in the hospital. The second time my head was a little dizzy. After that, they tied me up. I am one guy who can do nothing.

Feeling disempowered, this patient went on to explain that there was no hope for his future. “The future is not for me, no one can help me,” he remarked. In despair, he felt there was nothing he could do as his perspective and wishes were overridden.

The hospital has least restraint policy, “Restraint may be initiated only when the patient's behaviour or actions could result in harm to self or others, and interventions that maximize freedom have been attempted, and deemed unsuccessful. Whenever possible, the patient and/or substitute decision maker must be involved in the decision‐making process.” However, patients with dementia are often assumed as incapable of making care decisions so hospital staff would go to the family to seek opinion. Sometimes, family's perspective may not necessarily be the same as the patient's. In this case, the son did not want his father to take risk of falls and insisted on restraint use. The patient however had a good insights into the risks of being restraint and would rather have the freedom to walk. He explained, “My body and legs are getting weaker because I could not exercise when I am being tied up.”

Another patient expressed deep resentment about being denied of a pair of scissors. He felt strongly that his rights were being violated. He felt his voice was not heard or respected. Being a patient with a diagnosis of dementia constrained his ability to exercise citizenship rights. Patients were not allowed to have any scissors in the ward because staff believed it was too risky and patients might hurt themselves. In protest, “Tell the people who run the show that little things matter. It would be nice to be able to do things like having a small pair of scissors to cut things.” This patient told the researcher that he likes cutting interesting newspaper clippings, which was something he had always enjoyed to do all his life.

Other issues related to respecting rights that were raised by patients included their experiences of social exclusion and discrimination on the unit. One patient felt that due to changes in his cognitive function and the label of dementia, he was viewed and treated as a subclass on the ward. He said, “I want to be one of you guys… I don't have the freedom. And I swear it's not right. I just can't fathom the system. They have the rights over me.” He explained further: “Patients who are not with it, just don't have the freedom. If you're not with the freedom, then we might as well be dead.” In this case, the patient felt that a loss in freedom means losing humanity. It was evident that how others treated him impacted how he viewed himself. He called himself a loser:

I feel like being a loser! Yeah. It's a shame, you know, you guys have your freedom, and you know what, I have none. My door isn't locked. But your doors are locked. You can go out to eat. The only thing I get is from the buggy (kitchen cart). The freedom that I get is a piece of shit. Yeah, it shouldn't be like that, you know. I have no right. I can't even go out there and buy anything, like the small things, like going to a coffee shop. It's terrible to live one‐sided.

This patient gave a strong expression of his feeling of injustice and inequalities of power.

Social inclusion was also pertinent to the participants. Being recognised as a full citizen meant not only having rights for themselves but also involved having responsibilities and opportunities to help others. One patient suggested, “In the hospital, we are just a number, which does not mean much. Maybe it (working in the project) just makes you feel that you're contributing to people beside yourself. I think that we all have something in our mind that we should be able to say because we're not just only ourselves.”

All participants expressed that they appreciated being asked about their experience and views of the hospital environment. They felt that contributing their opinions and suggestions to improve the environment meant they were being respected, and their views mattered. They were excited about the interviews, video making and spoke highly about the experience. A patient described making video stories of her experience as being fun, and its process offered a positive distraction, which helped her to adjust to being in the hospital. Another patient said participated in the research work made her feel a useful member of the community and that she had done a good deed. At the same time, the participants clearly expressed that they expected that the new knowledge would become part of education and would inform actions in making improvements.

## 5 Discussion

The current study explored what and how specific environmental attributes might impact the care experiences of patients with dementia and what suggestions patients with dementia wished to see as improvements to the issues they identified. The analysis has shown that the relationships between environmental attributes and care experiences of patients with dementia are complex, with both the physical and social environments having significant impacts on the care experience of the participants. Participants told insightful stories about their experiences and persuasively described what mattered to them in the care environment of the hospital.

A place of enabling independence was a theme that showed up early and consistently across data in interviews and observations. Congruent with the literature, qualities of lighting, colour and objects were identified by the participants as pertinent factors in the physical environment supporting and hindering their feeling of independence. The data have shown that some participants faced difficulties with sensory overstimulation. Also, visual impairment could significantly decrease the stress thresholds. For improvement, participants suggested using shelving and storage to reduce clutter. They also indicated that colour might be used to reduce the risk of getting lost and make wayfinding easier. Fun and bright colours were preferred. Other studies have made similar recommendations (Chaudhury & Cooke, 2014; Karlin & Zeiss, 2006).

Sensory deprivation and boredom were also a common and paramount issue. Participants explained that a place that supports social interactions is essential for their well‐being. This is similar to another study in an acute psychiatric unit that found older patients with dementia perceived social connection through having things to do with others was essential to maintain self‐esteem and well‐being (Hung et al., 2014). The participants in this study suggested helpful strategies included creating activity space and comfortable areas for conversations. These strategies are in line with the recommendations written by scholars in dementia design (Andrews, 2013; Calkins, 2013).

Because dementia can affect a person's memory and communication skills, which can lead to feelings of insecurity, it becomes more important for them to be in places that feel emotionally safe. Participants explicated that a place of safety should afford opportunities to do familiar everyday activities such as going for walks. Overcrowded hallways and fast‐paced traffic led to apprehension, anxiety and psychological distress. This is similar to the findings of Edvardsson, Winblad, and Sandman (2008), who described feeling safe and cared for as central for older people in the hospital. They defined the therapeutic environment as constituted by the physical environment, the staff in the environment, as well as the general climate of care. Participants in this study mentioned that having nurses close by helped them feel safe. Other studies had reported similar results, that when nurses were right there, fear was decreased (Hung et al., 2014; Shattell, Hogan, & Thomas, 2005).

One of the important points that was clearly voiced by the patients in this study, though scarcely mentioned in the hospital environment literature, was the notion of respect for citizenship rights. The patient said that the restrictive environment not only meant a loss of freedom, but also a loss of their rights. Scholars in nursing and humanistic geography have written about the power of place in determining how a person may be with others (Casey, 2009; Liaschenko, Peden‐McAlpine, & Andrews, 2011). Casey (2009) argued that a place has the power “to direct and stabilize us, to memorialize and identify us, to tell us who and what we are in terms of where we are” (p. xv). Patients in this study strongly voiced how they were affected by environmental constraints and structures imposed by the hospital place. Their capacity to perform in the hospital environment was influenced by how they were accommodated by the physical environment, clinical structures and the social climate. This study offers preliminary first‐person insight of these issues; future research should further investigate the potential for supporting patient involvement in environment design and service development using a rights‐based approach (Kelly & Innes, 2013) and a participatory approach.

Interestingly, some of our results differ from those of Digby and Bloomer (2014), where the patients said the physical environment did not matter as long as the care was good, the noise was accepted as being normal, and the colours were not identified as being important. As a possible explanation, the setting they investigated was a new, modern and purpose‐built facility. In contrast, the setting of our study was of a traditional design having many challenging physical environmental features. In our study, the patients referred to the importance of colours, the clutter and the need for comfortable seating places. Our findings, which referenced the importance of colours, clutter and the need for comfortable seating places, are congruent with Bromley's (2012) suggestion that the aesthetics of the hospital environment are relevant to person‐centred care as the design decisions “send substantive messages about hospital priorities, power relations and moral values” (p. 1065).

As a final point of discussion, as we have noted above, action researchers emphasise the agenda of emancipatory politics, which requires careful connections between the methodology and the concerns of the population (Bradbury, 2015). Patients are experts of the illness and care experience. To gain real learning for responsive change, researchers and leaders need to be committed to “working with” patients in service evaluation and development. Our findings demonstrate that older patients with dementia have useful knowledge for contributing to service development. Instead of emphasising problems, their narratives have provided practical solutions for improving the hospital services. Our data also illustrated that some of the concepts brought up as highly valued by people with dementia were in clear conflict with what was considered as priorities by staff in the unit. What safety means to staff often trumps the perspective of patients.

### 5.1 Limitations

A limitation of this study is that patients who did not speak English were excluded. Another limitation is the fact that the investigation focused on public areas in the corridors. Future studies should include the bedroom and bathroom areas, which likely present other challenges. The views reported here were from the perspectives of patient participants. The views of frontline staff, physicians and the organisational leaders will be reported in another paper. For a redesign of healthcare settings, main challenges for research are not only about identifying the needs of the patient group or the team of staff, but also negotiating an integration of all these needs into the already existed built environment. It requires a collaborative approach that involves all stakeholders and methodology that helps to address the different environmental domains and perspectives in a holistic way (Iedema et al., 2015).

## 6 Implications and conclusions

This research provides empirical support to the importance of creating positive, supportive environments in hospitals by paying attention to the physical environment and social processes in the place. There are key implications for nursing practice, service development and future research. Firstly, nurses working in acute care are well positioned to take an active and leading role in bringing patients’ voices forward in everyday practice. Identifying the specific needs that patients with dementia experience in the hospital environment could inform practical strategies to provide more responsive care. Our results indicate that patient stories captured in videos permitted a rich and more nuanced description of patients’ experiences than what is possible with quantitative measures. Thus, filming short videos and viewing them in staff reflexive sessions has great potential to offer easily accessible advantages that are valuable for team learning in the local context. Future research should examine the challenges, risks and benefits of using videos for practice development.

Secondly, leaders who are responsible for service development and hospital design or redesign need to recognise the problems voiced by patients about the hospital environment. Participants in this study explained how environmental features restricted patients’ agency to maintain health and well‐being and they offered simple, practical and inexpensive solutions to improve the existing environment. Also, the physical features were only a part of the hospital environment which is a complex combination of multicomponents, including nurses’ availability, dementia knowledge and care practice. Therefore, a multipronged approach is required to create an optimised care environment in acute care settings.

Thirdly, future research is needed to better understand how to best support involvement of patients with dementia in making service improvement and respecting their rights in making healthcare decisions. While the findings of the small sample cannot be generalised, we can learn a great deal from their direct perspective and their involvement in the research. In this study, the technique of go‐along interviews was used. We found the combined conversational interview with the use of environmental cues in the immediate context was effective in supporting patients with dementia to express their views. More research is needed to further investigate how go‐along interviews may serve as a useful tool in dementia research in terms of meeting the need to examine how physical and social dimensions of the environment might interact and influence the person in organic ways.

To conclude, this study reveals how the hospital unit is a complex system with environmental components that interact to influence the experiences of patients with dementia. For the patients with dementia who participated in this study, a good hospital environment needs to be a place enabling independence, a place of safety, a place that supports social interactions and a place of respect. The participants highlighted in particular the challenges they faced in disempowerment and a loss of citizenship rights. We call for political efforts in research and practice to seek a shift away of seeing patients with dementia as passive to more active citizens, who have rights to participate in research and projects aiming to redesign healthcare services that directly affect them.

7

**Implications for practice**

- Patient involvement in redesigning hospital environment will provide a way to integrate values of patient experiences into service improvement.
- Hospital leaders and practitioners need to view patients with dementia as partners ‐ listen and have a conversation with patients with dementia.
- Seeing patients as partners means willingness to invest resources for patient engagement ‐ video stories told by patients with dementia is a possible way.

## Contributions

Study design: LH designed the study with guidance from AP, HC, PR; Data analysis: LH, AP, HC, PR, JT, DB; Manuscript preparation: LH wrote the first draft, All authors reviewed and provided input for revisions.
